# Association of Vitamin D Metabolites With Embryo Development and Fertilization in Women With and Without PCOS Undergoing Subfertility Treatment

**DOI:** 10.3389/fendo.2019.00013

**Published:** 2019-01-29

**Authors:** Thomas Keith Cunningham, Victoria Allgar, Soha R. Dargham, Eric Kilpatrick, Thozhukat Sathyapalan, Stephen Maguiness, Haira R. Mokhtar Rudin, Nour M. Abdul Ghani, Aishah Latiff, Stephen L. Atkin

**Affiliations:** ^1^Hull IVF Unit, Women and Children's Hospital, Hull Royal Infirmary, Hull, United Kingdom; ^2^Centre for Diabetes and Metabolic Research, Hull York Medical School, University of Hull, Hull, United Kingdom; ^3^Department of Statistics, Hull York Medical School, University of Hull, Hull, United Kingdom; ^4^Weill Cornell Medicine Qatar, Doha, Qatar; ^5^Sidra Medical and Research Centre, Doha, Qatar; ^6^Antidoping Laboratory Qatar, Doha, Qatar

**Keywords:** vitamin D, vitamin D epimers, vitamin D metabolites, fertilization rates, PCOS

## Abstract

**Objective:** The relationship between fertilization rates and 1,25-dihydroxyvitamin D (1,25(OH)_2_D_3_), 25-hydroxyvitamin D2 (25(OH)D_2_), 25-hydroxyvitamin D3 (25(OH)D_3_), 24,25-dihydroxyvitamin D (24,25(OH)_2_D_3_), and 25-hydroxy-3epi-Vitamin D3 (3epi25(OH)D_3_) concentrations in age and weight matched women with and without PCOS was studied.

**Methods:** Fifty nine non-obese women, 29 with PCOS, and 30 non-PCOS undergoing IVF, matched for age and weight were included. Serum vitamin D metabolites were taken the menstrual cycle prior to commencing controlled ovarian hyperstimulation.

**Results:** Vitamin D metabolites did not differ between PCOS and controls; however, 25(OH)D_3_ correlated with embryo fertilization rates in PCOS patients alone (*p* = 0.03). For all subjects, 3epi25(OH)D_3_ correlated with fertilization rate (*p* < 0.04) and negatively with HOMA-IR (*p* < 0.02); 25(OH)D_2_ correlated with cleavage rate, G3D3 and blastocyst (*p* < 0.05; *p* < 0.009; *p* < 0.002, respectively). 24,25(OH)_2_D_3_ correlated with AMH, antral follicle count, eggs retrieved and top quality embryos (G3D3) (*p* < 0.03; *p* < 0.003; *p* < 0.009; *p* < 0.002, respectively), and negatively with HOMA-IR (*p* < 0.01). 1,25(OH)_2_D_3_ did not correlate with any of the metabolic or embryo parameters. In slim PCOS, 25(OH)D_3_ correlated with increased fertilization rates in PCOS, but other vitamin D parameters did not differ to matched controls.

**Conclusion:** 3epi25(OH)D_3_, 25(OH)D_2_, and 24,25(OH)_2_D_3_, but not 1,25(OH)_2_D_3_, were associated with embryo parameters suggesting that vitamin D metabolites other than 1,25(OH)_2_D_3_ are important in fertility.

## Introduction

Polycystic ovarian syndrome (PCOS) is one of the most common endocrine disorders amongst women of reproductive age affecting 9–21% of the female population and is the main cause of anovulatory infertility (1). It is associated with clinical and biochemical hyperandrogenism, and insulin resistance (IR) in PCOS is associated with obesity, type 2 diabetes, and hypercholestrolemia (2). Vitamin D levels are low in 67–85% of women with PCOS (3), which are suggested to exacerbate IR and the free androgen index (FAI) in PCOS (4, 5). IR itself is both independent of and exacerbated by obesity and is present in 65–80% of women with PCOS (6) and may be improved by vitamin D replacement (7). In a recent meta-analysis, it was shown that in weight matched PCOS women, vitamin D was negatively predicted by weight hip ratio, glucose and LH (8).

Vitamin D deficiency has become the most common nutritional deficiency throughout the world (9). Studies of sub-fertile women have demonstrated that vitamin D deficiency is present in between 58 and 91% of cases (9–12). Obesity can exacerbate vitamin D deficiency, as a result of decreased bioavailability from cutaneous and dietary sources because of deposition in the body fat compartments (13).

**Figure 1:**
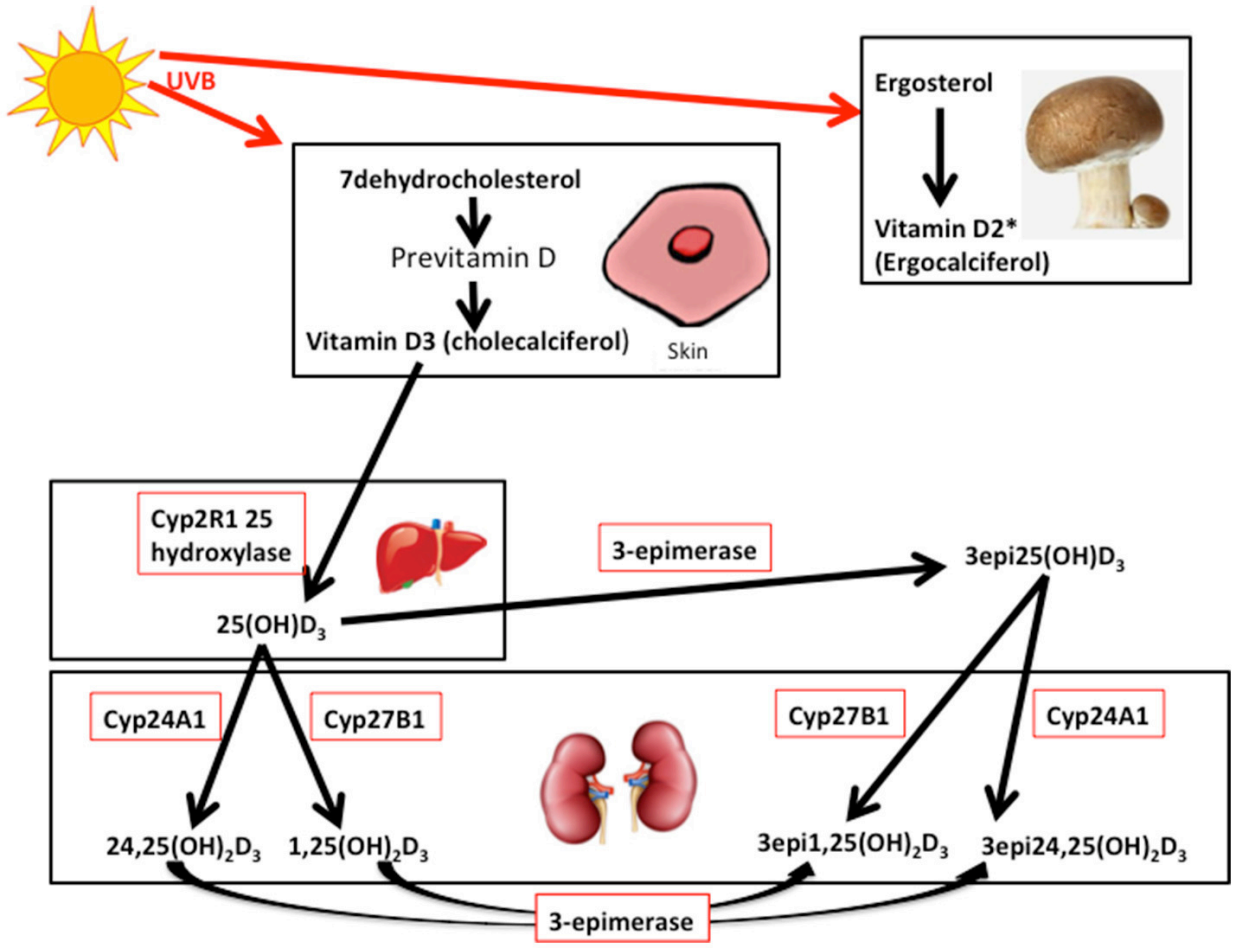
7-dehydrocholesterol in the skin is converted to previtamin D3 and then is thermally isomerize to vitamin D3. Transport of vitamin D_3_ from the skin to the liver is via the Vitamin D binding protein (DBP) transports 25 hydroxy vitamin D (25(OH)D_3_) to the kidney. 25(OH)D_3_/DBP is filtered by the glomeruli and 25(OH)D_3_ is taken up into the tubular cells, following DBP binding to megalin, a transmembrane protein. 25(OH)D_3_ undergoes a second hydroxylation step by the 1-alpha-hydroxylase Cyp27B1, converting to the active 1α,25 (OH)_2_D_3_, whilst 24 hydroxylase Cyp27A1 converts to 24,25(OH)_2_D_3._ Keratinocytes contribute to the 3-epimerase activity converting 25(OH)D_3_ to 3-epi-25(OH)D_3_, and 1α,25 (OH)_2_D_3_ to 3-epi-1α,25 (OH)_2_D_3_, but the exact sites of activity remain unknown. 3epi25(OH)D_3_ is equally converted by Cyp27A1 and Cyp27B1 as 25(OH)D_3_. ^*^Vitamin D_2_ derived from yeasts and fungi (mushrooms) is converted to 25(OH)D_2_ in the liver and to 1,25(OH)_2_D_2_ and 24,25(OH)_2_D_2_ in the kidney (adapted from 14, 16, and 21). It is unclear if the 3-epimers may be back converted.

Vitamin D3 (cholecalciferol) is endogenously produced or taken as a dietary supplement, whist vitamin D2 (ergocalciferol) is derived from the diet (primarily from mushrooms and fungi), though both are hydroxylated to 25(OH)D_3_ or 25(OH)D_2_ by multiple 25-hydroxylases (14, 15) (Figure 1). 25(OH)D is transported to the kidney and converted to either the active 1,25-dihydroxyvitamin D (1,25(OH)_2_D_3_) by 1 alpha hydroxylase, or to 24,25-dihydroxyvitamin D (24,25(OH)_2_D_3_), that is also active, by the 24 alpha hydroxylase, in the renal tubular and other cells widely in the body (Figure 1) (16). It has been recently reported that extrarenal tissues may also convert 25(OH)D to 1,25(OH)_2_D (17). 1,25(OH)_2_D binds to the vitamin D receptor (VDR) subsequently heterodimerizes with the retinoid X receptor for its action that may be effected in several hours (16); however, a more rapid action has been reported with binding membrane VDR or through the 1,25D_3_-membrane-associated, rapid response steroid-binding protein receptor with activation of protein kinases A and C (18). Vitamin D receptors have been located within structures of the female reproductive tract, including the ovary and endometrium (19, 20).

Vitamin D_2_ is derived from the diet as ergocalciferol that has lower binding efficacy to VDR resulting in greater serum clearance, limiting the formation of 25(OH)D_2_, though 1,25(OH)_2_D_2_ has a high an affinity for VDR as 1,25(OH)_2_D_3_ (14, 16). In the United States and other countries vitamin D2 is available both as a supplement and as a pharmaceutical to treat vitamin D deficiency.

3- epimerase isomerizes the C-3 hydroxy group of the natural vitamin D from the α to the β orientation leading to 3epi25(OH)D_3_ (14, 21) that may be measured inadvertently whilst measuring 25(OH)D_3_ (22). 3epi25(OH)D_3_ is thought to be less potent physiologically as 25(OH)D_3_, and 1,25(OH)_2_-3-epi-D_3_ has less affinity to VDR thus less biologically active; however, the 3-epimer may be as potent as 1,25(OH)_2_D_3_ in other circumstances such as PTH suppression (14, 23) however, data is sparse on the biological potency of the C3 epimers.

Two IVF cohort studies have suggested that clinical pregnancy rates were significantly lower in women who were vitamin D deficient (24, 25), but no differences in the embryological data have been associated with 25(OH)D_3_ levels (25). However, it remains unknown if there is a relationship of baseline 1,25(0H)_2_D_3_, 25(OH)D_3_, 25(OH)D_2_, 24,25(0H)_2_D_3_, or its epimer 3epi25(OH)D_3_ to fertilization in non-obese PCOS subjects when age and weight are matched to control subjects, and therefore this study was undertaken.

## Materials and Methods

This prospective cohort study was performed within the Hull IVF Unit, UK following approval by the Yorkshire and The Humber NRES ethical committee, UK and all gave their written informed consent. The PCOS subjects were recruited using the revised 2003 criteria (26), namely any 2 out of 3 criteria were met; menstrual disturbance (oligo or amenorrhoea), clinical and/or biochemical signs of androgenism and polycystic ovaries on ultrasound, with the exclusion of other conditions. All women were on folic acid 400 mcg daily but no other medication. Exclusion criteria were patients with diabetes, renal or liver insufficiency, acute or chronic infections, systemic inflammatory diseases, age < 20, age >45, known Immunological disease.

### Sample Collection

A fasting blood sample was taken in the luteal phase of the cycle before commencing IVF treatment. The bloods were centrifuged at 3,500 g for 15 min and placed into aliquots and frozen at −80°C until analysis. The bloods were analyzed for FSH (Architect analyser, Abbott laboratories, Maidenhead, United Kingdom), SHBG, insulin (DPC Immulite 200 analyser, Euro/DPC, Llanberis United Kingdom), and plasma glucose (Synchron LX20 analyser, Beckman-Coulter, High Wycombe, United Kingdom). Free androgen index (FAI) was calculated by dividing the total testosterone by SHBG, and then multiplying by one hundred. Insulin resistance (IR) was calculated using the homeostasis model assessment (HOMA-IR). Serum vitamin D levels and testosterone were quantified using isotope-dilution liquid chromatography tandem mass spectrometry (LC-MS/MS). Vitamin D metabolites (1,25(OH)2D3), 25(OH)D2, 25(OH)D3, 24,25(OH)2D3 and 3epi25(OH)D3 and three labeled internal standards (d6-25(OH)D3, d6-1,25(OH)2D3 and d6-3-epi-25(OH)D3) were simultaneously extracted from 250 μL serum using supportive liquid-liquid extraction and Diels-Alder derivatization prior to LC-MS/MS analysis. Chromatographic separations were achieved using Hypersil Gold C18 column (150 × 2.1 mm; 1.9 μ) at flow rate 0.2 ml/min, operated in Electrospray Ionization (ESI) positive mode and analyzed by multiple reaction monitoring (MRM) method. The limit of quantification (LOQ) for 1,25(OH)2D were 10 pg/mL, 3-epi-25(OH)D, and 24,25(OH)2D were 50 pg/mL while 25(OH)D3 and 25(OH)D2 were 0.5 and 0.25 ng/mL, respectively. All methods employed were performed in accordance with the relevant guidelines and regulations.

All patients underwent a standard IVF antagonist protocol. The patients commenced their rFSH stimulation on day 2 of their menstrual cycle using either Merional (Pharmasure) or Gonal-F (Merck Serono). A GnRH antagonist (Cetrotide: Merck Serono) was used to prevent a premature LH surge.

The patients underwent ultrasound scans from day 7 to observe the ovarian response to stimulation and were repeated every 48 h. The scans were used to measure the diameters of the follicles thus observing response and follicle numbers. Final maturation was triggered when two or more leading follicles were ≥18 mm using human chorionic gonadotrophin [hCG, Pregnyl (Merck Sharp and Dohme)].

Transcervical embryo transfer was performed and embryos were classified using standard criteria (27) at the cleavage stage (day 2–3 after egg collection) and for blastocyst stage (day 5–6 after egg collection). Top Quality embryos on Day 3 as per Alpha Consensus (28). Embryo transfers were performed on either day 3 or ideally at day 5 (blastocyst) to give the best chance for implantation as this timing is similar compared to natural cycle embryos moving into the uterus.

### Data Analysis and Statistics

Statistical analysis was performed using SPSS (v22, Chicago, Illinios). Descriptive data is presented as mean ± SD for continuous data and n (%) for categorical data. *t*-tests or Mann Whitney tests were used to compare means/medians where appropriate, and associations used Pearson's correlation or Spearman's correlation as appropriate. A *p* < 0.05 was considered to indicate statistical significance. There was no comparative study on which to base a formal power calculation; therefore, power and sample size for a pilot study was performed (29); therefore, to account for a minimum of 20 degrees-of-freedom to estimate effect size and variability a minimum of 25 patients per group were required to allow covariate adjustment.

## Results

Baseline characteristics of the 59 patients are shown in Table 1 where is can be seen that patients were non-obese, age, and weight matched. There were significant differences in ovarian reserve parameters antral follicle count (AFC) and anti-Mullerian Hormone, (AMH), and androgen status between the groups, however there was no significant difference in fasting insulin, HOMA-IR or the vitamin D metabolites (Table 1).

**Table 1:** Mean demographics and biochemical data.

	**Control (*n* = 30)**	**PCOS (*n* = 29)**	***p*-value**
	**Mean data (±S.D.)**	**Mean data (±S.D.)**	
Age	32.6 ± 4.7	30.9 ± 4.8	0.14
BMI	25.5 ± 3.6	26.0 ± 3.8	0.56
Menarche	13.0 ± 2.0	13.0 ± 1.1	0.99
Anovulatory	5	25	0.0001^***^
Duration of subfertility	3.9 ± 1.8	3.4 ± 1.6	0.84
Total antral follicle count	17.2 ± 6.8	38.4 ± 17.8	0.0001^***^
Fasting insulin (mIU/ml)	7.68 ± 4.0	8.13 ± 4.7	0.69
Fasting glucose (mmol/L)	4.81 ± 0.4	4.62 ± 0.4	0.06
HOMA-IR	1.71 ± 1.0	1.72 ± 1.0	0.97
SHBG	110.9 ± 82.4	63.9 ± 49.8	0.01^*^
Testosterone (mmol/L)	0.8 ± 0.4	1.4 ± 0.8	0.0004^***^
Free androgen index	1.35 ± 0.6	4.21 ± 2.9	0.0001^***^
25-hydroxyvitamin D3 (ng/mL)	46.2 ± 23.5	54.0 ± 27.4	0.24
25-hydroxyvitamin D2 (ng/ml)	0.5 ± 0.3	0.6 ± 0.5	0.73
1,25-dihydroxyvitamin D3	0.03 ± 0.02	0.04 ± 0.2	0.63
24R,25-dihydroxyvitamin D3	0.8 ± 0.5	1.3 ± 0.6	0.003^**^
3-epi-25-hydroxyvitamin D3	0.4 ± 0.4	0.7 ± 1.2	0.89

* p < 0.01,

** p < 0.001,

*** *p < 0.0001*.

There was a correlation between the levels of 25(OH)D_3_, and embryo fertilization rates in PCOS patients (*r* = 0.44; *p* = 0.03) that were not seen in the control group. However, between the PCOS and control groups there were no differences for any of the metabolic or embryo parameters for 25(OH)D_2_, 24R,25(OH)_2_D_3_, 1,25(OH)_2_D_3_, or 3epi25(OH)D_3_. When all of the subjects were combined there was a correlation between the levels of 25(OH)D_2_ and cleavage rate (*r* = 0.31; *p* = 0.05), G3D3 (*r* = 0.40; *p* = 0.009) and blastocyst (*r* = 0.40; *p* = 0.022); there was a correlation between 3epi25(OH)D_3_ and fertility rate (*r* = 0.33; *p* < 0.04) and a negative correlation with HOMA-IR (*r* = −0.33; *p* < 0.02); 24R,25(0H)_2_D_3_ correlated with AMH (*r* = 0.1; *p* = 0.03) antral follicle count (*r* = 0.2; *p* = 0.003), eggs retrieved (*r* = 0.14; *p* = 0.009) and G3D3 (*r* = 0.22; *p* = 0.002), and negatively with HOMA-IR (*r* = −0.07; *p* < 0.01). There was no correlation of the active 1,25(0H)_2_D_3_ with any of the metabolic or embryo parameters.

There was a correlation between the levels of 25(OH)D_3_ with both 24R,25(OH)_2_D_3_ and 3epi25(OH)D_3_ (*r* = 0.91, *p* < 0.001; *r* = 0.35, *p* < 0.015, respectively.

As a cohort, 25-hydroxyvitamin D levels were low did not differ between the controls and the PCOS group. The Endocrine Society defines vitamin D deficiency, insufficiency and replete as (≤20 ng/mL, 20–30 ng/mL and ≥30 ng/mL, respectively (30) that was reflected in controls and PCOS as, deficient, 51 vs. 41%; insufficient, 33 vs. 35%; deficient, 10 vs. 24%.

IVF cycle characteristics are represented in Table 2 showing that the PCOS group had significantly greater numbers of follicles aspirated and eggs retrieved compared to the controls, and the mean fertilization and cleavage rates were significantly higher for the PCOS group, though embryos quality did not differ.

**Table 2:** Mean outcome data for stimulated ovarian cycle for Control and PCOS groups. G3D3: Top Quality embryos on Day 3 as per Alpha Consensus (28).

	**Control (*N* = 30)**	**PCOS (*N* = 28)**	***p*-value**
	**Mean (±S.D.)**	**Mean (±S.D.)**	
Endometrium at oocyte retrieval	10.31 ± 1.78	10.72 ± 2.06	0.42
Follicles aspirated	11.47 ± 5.11	15.96 ± 5.30	0.002^**^
Eggs retrieved	8.47 ± 5.08	11.29 ± 5.02	0.04^*^
Fertilization	4.82 ± 2.65	8.43 ± 3.87	0.0003^***^
Cleavage	4.68 ± 2.72	7.26 ± 4.40	0.01^*^
G3D3	3.00 ± 2.29	4.17 ± 3.47	0.16
Blastocyst	1.46 ± 1.77	2.91 ± 3.01	0.05
PDT	11	10	0.86
Clinical pregnancy	10	7	0.24

* p < 0.01,

** p < 0.001,

*** *p < 0.0001*.

There was a significantly negative correlation between SHBG and 25-hydroxyvitamin D3 in the PCOS subjects, however after adjusting for BMI, SHBG was not significantly associated with 25-hydroxyvitamin D3.

## Discussion

This study has shown that 25(OH)D_3_ was associated with higher fertility rates in PCOS compared to non-obese, age, and weight matched control subjects, but that this was not seen for the other vitamin D metabolites. This was surprising given that there was no difference in the 25(OH)D_3_ levels between the PCOS and control group; however, it is recognized that ova in PCOS may be at a less mature stage compared to normal and therefore there is a possibility that they may be more 25-hydroxyvitamin D responsive to allow those ovum within the stimulated follicles to reach a more mature stage prior to ovum retrieval, resulting in a greater capability to achieve fertilization. PCOS women typically produced more poor quality oocytes, with lower fertilization, cleavage and implantation rates (31, 32). The impaired oocyte maturation and resultant embryonic developmental competence in PCOS women is possibly due to the abnormal endocrine/paracrine functions and the environment within the follicle at the time of folliculogenesis (33, 34). No differences in the embryological data for 25(OH)D_3_ were found, in accord with others (25). It may have been speculated that the active 1,25(OH)_2_D_3_ may have a greater influence on fertility at higher levels, but this was not seen in this study, with no correlation with fertilization or embryo data. Vitamin D is involved in the regulation of AMH and FSH gene expression (31, 35), and high dose 25(OH)D_3_ has been shown to increase serum AMH levels in vitamin D insufficiency (36). In this study only the metabolite 24R,25(OH)_2_D_3_ correlated with AMH and antral follicle count; 24R,25(OH)_2_D_3_ is an active metabolite [It can be converted to 1,24,25-trihydroxyvitamin D3 through the C24 oxidation pathway (37)] as it has been shown to induce non-genomic signaling pathways and suppresses Apo A-1 in hep G cells (38), may have a physiological role in the growth plate formation (14), therefore a direct effect on the ovary cannot be excluded. However, 24,25 dihydroxyvitamin D is associated with blood levels of 25-hydroxyvitamin D and given that both were very significantly associated it is unlikely that 24R,25(OH)_2_D_3_ was having a unique biological effect and indeed was dependent on serum 25(OH)D_3_ levels.

There was a positive association for an increase in overall fertility rate with the 3epi25(OH)D_3_ that was not seen for the other vitamin D metabolites. Little is known about the epimers of vitamin D, with the assumption that they are biologically less potent (21, 23), but whilst that is likely for bone metabolism it may not be the case for ovarian effects. Epimers are compounds that have identical structure (and therefore identical molecular weight) with the exception of a stereochemical difference at one site. Other than the measurement of 3epi25(OH)D_3_ to enable the more accurate determination of 25(OH)D_3_, in children where they have been shown to be higher, there has been little research done on the C3 epimers (39) in order to know the significance of this observation on fertility, but it is of interest to note that serum lipids were discrepant for 25(OH)D epimeric forms suggesting a differential effect (40). However, 3epi25(OH)D_3_ is associated with blood levels of 25-hydroxyvitamin D and given that both were very significantly associated it is unlikely that 3epi25(OH)D_3_ was having a unique biological effect and indeed was dependent on serum 25(OH)D_3_ levels.

Whole group analysis for 25(OH)D_2_, but not 25(OH)D_3_ was positively associated with increased cleavage rate, G3D3 and blastocyst, though overall embryo quality did not differ. Whilst vitamin D3 supplements are better than vitamin D2 to raise vitamin D levels (41, 42), their biological effects may not be the same in different systems (43), and their differential effect in the ovary needs to be clarified. The role of vitamin D in fertilization remains controversial and the specific roles of 25-hydroxyvitamin D levels largely unknown. Observational studies have reported vitamin D levels within serum and follicular fluid to be highly correlated and that those with higher serum and follicular fluid levels of vitamin D had significantly higher clinical pregnancy rates (12). Other studies have found no correlation between serum and follicular fluid levels of vitamin D and IVF outcomes (10, 35, 44). Conversely, two cohort studies comparing serum vitamin D levels and pregnancy rates in women undergoing fresh IVF showed that clinical pregnancy rates were significantly lower in women who were vitamin D deficient (24, 25). Given the controversy, the implication of this data is that in slim PCOS women undergoing IVF that their vitamin D status should be determined, vitamin D replacement undertaken for those deficient prior to IVF may be of benefit, or at least do not harm, until future clarification becomes available.

These data suggest that vitamin D deficiency may not be a homogeneous entity but rather may depend on the different vitamin D metabolites present giving resultants effects, and therefore may account for the heterogeneity and controversy surrounding vitamin D deficiency effects and the response to replacement (42, 45). Whilst the effects of vitamin D and its metabolites on bone and calcium metabolism are well-known, vitamin D metabolite effects on other systems may not be directly equipotent for each metabolite or comparable. For example 1,25(OH)_2_-3-epi-D_3_ may be biologically less active than 1,25(OH)2D3, for increasing calcium, but may have greater PTH suppression (39). However, the kidneys produce 1,25-dihydroxyvitamin D for regulating calcium and bone metabolism (46). Therefore, measuring a blood level of 1,25-dihydroxyvitamin D may not reflect its other biologic functions, but rather local production of 1,25-dihydroxyvitamin D may have its major benefit (47). Our observation that higher blood levels of 25-hydroxyvitamin D are related to the outcome measures could also be due to the higher substrate availability for the local production of 1,25-dihydroxyvitamin D rather than 25-hydroxyvitamin D having a direct effect.

When weight and aged matched then there was no difference in vitamin D metabolite levels between PCOS and normal women. These results differ from previous studies that found Vitamin D deficiency to be more common in PCOS subjects (48); however, in this study the PCOS patients were specifically non-obese and it is well-recognized that vitamin D levels fall in obesity that may account for this observation (6). Negative correlations with HOMA-IR were seen for both 24R,25(OH)_2_D_3_, and 3epi25(OH)D_3_, but no association was seen for either 25(OH)D_2_ or 25(OH)D_3_, suggesting that an association with insulin resistance may depend on the metabolites present. There were no correlations between testosterone or oocyte quality in the PCOS patients in this study These observations are in accord with some studies, though not with others (4, 49); however, in those reported studies patients were not intentionally weight matched. High dose 25(OH)D_3_ replacement was not associated with an improvement in insulin sensitivity in PCOS subjects (50).

This study was specifically designed to look at the relationship of vitamin D levels with fertilization rates and therefore was not powered to look at pregnancy rates that would require a much larger sample population. Furthermore, the overall sample size is small and further work is needed, particularly to determine if these findings are true for the differing PCOS phenotypes within the Rotterdam criteria. However, the strengths of this study were that the patients were age and weight matched from the same ethnic background and that all of the vitamin D metabolites were measured by state of the art methods.

In conclusion, non-obese age and weight matched PCOS women showed that 25(OH)D_3_ was associated with the fertilization rate, compared to controls; however, vitamin D metabolites were associated with embryology parameters and HOMA-IR, suggesting a possible relationship between differing vitamin D metabolites, oocyte maturation and insulin sensitivity in non-obese PCOS patients.

## Author Contributions

TC was involved in the study design, acquisition of data, analysis and interpretation of data, and paper drafting. HM, NA, and AL were involved in vitamin D analysis and drafting the manuscript. VA and SD were involved in analysis and interpretation of data, and paper drafting. SA, EK, SM, and TS were involved in the study design, supervision, paper drafting, and contributed to the interpretation of the data. All authors read and approved the final manuscript.

### Conflict of Interest Statement

The authors declare that the research was conducted in the absence of any commercial or financial relationships that could be construed as a potential conflict of interest.
